# Aqueous extract of *Monodora myristica* ameliorates cadmium-induced hepatotoxicity in male rats

**DOI:** 10.1186/s40064-016-2228-z

**Published:** 2016-05-17

**Authors:** Babatunji Emmanuel Oyinloye, Abiola Fatimah Adenowo, Foluso Oluwagbemiga Osunsanmi, Bolajoko Idiat Ogunyinka, Sarah Onyenibe Nwozo, Abidemi Paul Kappo

**Affiliations:** Biotechnology and Structural Biology (BSB) Group, Department of Biochemistry and Microbiology, University of Zululand, KwaDlangezwa, 3886 South Africa; Department of Biochemistry, College of Sciences, Afe Babalola University, PMB 5454, Ado-Ekiti, 360001 Nigeria; Nutritional and Industrial Research Laboratories, Department of Biochemistry, Faculty of Basic Medical Sciences, College of Medicine, University of Ibadan, Ibadan, 200002 Nigeria

**Keywords:** Cadmium, Hepatic enzyme, Livolin forte, *Monodora myristica*, Oxidative stress

## Abstract

In recent years, indigenous medicinal plants exhibiting diverse biological activities have been explored in the amelioration of hepatotoxicity. This study investigates the protective effect of *Monodora myristica* (MM) on cadmium-induced liver damage in experimental animals. Male Wistar albino rats were maintained on 200 mg/L cadmium: Cd (Cd as CdCl_2_) in the animals’ main drinking water to induce hepatotoxicity. Added to this, the animals received aqueous extracts of MM at a dose of 200 or 400 and 20 mg/kg bw of Livolin forte (LF) for 21 days. At the end of the experiment, levels of serum enzyme biomarkers (alanine transaminase, alkaline phosphatase and aspartate transaminase) as well as total cholesterol (TC), triacylglyceride (TG) and malondialdehyde were significantly raised in the cadmium treated groups. Conversely, cadmium treatment elicited noticeable decrease in hepatic enzymatic and non-enzymatic antioxidants (reduced glutathione: GSH, catalase: CAT, superoxide dismutase: SOD). Co-treatment with MM at varying doses as well as LF considerably decreased the elevated levels of the serum biomarkers as well as TC, TG and malondialdehyde in the cadmium-treated groups in a dose dependant manner. Additionally, MM exhibited reversal potential on cadmium-toxicity at the tested doses as its administration was accompanied by a pronounced increase in GSH, SOD, and CAT levels. Histopathological results were parallel to these findings. These results demonstrates that aqueous extracts of MM is effective in the amelioration of hepatic damages arising from cadmium-induced toxicity, indicating that the antioxidant bio-constituents of MM play an important role in the prevention of liver toxicity possibly by inhibiting bioaccumulation of free radicals in animal models.

## Background

Bioaccumulation of cadmium; a ubiquitous non-degradable environmental pollutant that enters the food chain is an issue of severe global concern. Its environmental accumulation is due to its increased industrial usage in mining, electroplating, dyeing, paints, just to mention a few, as well as its occurrence in agricultural fertilizers (Renugadevi and Prabu 2009; Newairy et al. 2007). The extensive environmental distribution of this heavy metal prompted an increased attention as regards its biological effects and toxicity. Some documented deleterious effect of cadmium toxicity includes hepatocellular damage, testicular atrophy, hypertension, renal dysfunction, anaemia and injury to the central nervous system (Jeyaprakash and Chinnaswamy 2005; Vicente-Sánchez et al. 2008).

Cadmium induced toxicity in living systems may be due to a rise in lipid peroxidation, which could be accredited to changes in antioxidant defence systems including the enzymes thioredoxin reductase, glutathione peroxidase and reduced glutathione (GSH), which generally offers protection to living systems from toxicity due to free radicals (Newairy et al. 2007). Medicinal plants are acknowledged to have antioxidant activities because they are rich in several antioxidant molecules. Hence, in order to combat cadmium induced hepatic injury; medicinal plants might be appropriate due to their relative availability, low cost and minimal side effects. *Monodora myristica* is a tropical plant that belongs to the *Annonaceae* family; it is also called calabash, Jamaica or African nutmeg. This less-studied and greatly under-exploited plant is extensively distributed in Africa, Asia, Australia as well as Central and South America (Omobuwajo et al. 2003).

African nutmeg is a berry, which grows well in African evergreen forests where it is widely used as a condiment for different delicacies. Traditionally, it is used to cure sores from guinea worm infections, constipation, stomach-ache and headache as well as to stop intra-uterine bleeding in women after child birth. The seed oil is also useful as a carminative and for scenting perfumes and soaps (Burubai et al. 2008; Dada et al. 2013; Ekeanyanwu et al. 2010; Ojiako et al. 2010). Additionally, the root is munched to mitigate toothaches and arthritis and is also utilized in the management of anaemia, haemorrhoids as well as sexual weakness (Erukainure et al. 2012). Previous studies have reported the antioxidant properties of *M. myristica* seeds (Erukainure et al. 2012; Moukette et al. 2015). This minty smelling seed has also been investigated to possess cholesterol lowering activity (Nwozo et al. 2015), anti-sickling activity (Nwaoguikpe and Uwakwe 2008), antimicrobial activity (Esekhiagbe et al. 2009) as well as anthelmintic activity (Ekeanyanwu and Etienajirhevwe 2012).

This study was therefore designed to investigate the protective impact of *M. myristica* on cadmium-induced hepatic damage in experimental animal models.

## Results

### Phytochemical constituents

Phytochemical screening revealed the presence of alkaloids, saponin, tannins, flavonoids, cardiac glycosides and phenols in varying quantity in the aqueous extract of *M. myristica* seeds (Table 1).

**Table 1:** Results of the phytochemical screening of *M. myristica*

Phytochemicals	Alkaloids	Saponins	Tannins	Flavonoids	Cardiac glycosides	Phenols
Aqueous extracts	++	++	+	+++	++	+++

+, present in trace; ++, moderately present; +++, abundantly present

### Effect of *M. myristica* on the levels of liver marker enzyme activities

There was an elevation (P < 0.05) in the activities of hepato-specific enzymes; ALP, ALT and AST in the cadmium treated group (G2) when compared with the negative control (G1). Treatment with MM at doses 200 and 400 mg/kg bw showed a significant reduction (P < 0.05) in the levels of these enzymes. The observed reduction is comparable with the Livolin forte treated group (Table 2).

**Table 2:** The effect of *M. myristica* of the levels of liver marker enzyme activities

Treatment group	ALP activity (U/L)	ALT activity (U/L)	AST activity (U/L)
G1	110.37 ± 0.75^b^	115.61 ± 1.21^b^	121.25 ± 3.78^a,c^
G2	149.89 ± 0.44^a^	156.50 ± 1.80^a^	181.17 ± 5.20^b^
G3	120.04 ± 0.19	139.01 ± 3.79	169.82 ± 3.43^a^
G4	109.59 ± 0.61	104.12 ± 2.63	136.20 ± 1.57
G5	98.62 ± 0.32^a,b^	108.27 ± 0.13	127.27 ± 0.17

Values shown are mean ± SD (n = 6). Mean differences are significant (P < 0.05) when compared with: ^a^G1 (control group), ^b^ G2 (cadmium only)

### Effect of *M. myristica* on the levels of on MDA, TG and cholesterol

There was a significant increase (P < 0.05) in the levels of MDA, TG and cholesterol in the cadmium treated group (G2) when compared with the negative control (G1); however, treatment with the different doses of the aqueous extract prompted a significant reduction (P < 0.05). The observed reduction is similar to the results obtained for the standard drug group (Table 3).

**Table 3:** The effect of *M. myristica* on hepatic MDA, serum triglyceride and cholesterol levels

Treatment group	MDA (nmole/mg/protein)	Triglyceride (mg/dL)	Cholesterol (mg/dL)
G1	38.93 ± 0.96^b^	124.62 ± 2.23^b,c^	87.22 ± 5.60^b^
G2	70.66 ± 1.77^a^	175.11 ± 1.05^a^	173.20 ± 1.35^a^
G3	51.75 ± 2.32	154.23 ± 3.14^a^	111.20 ± 8.14^a^
G4	47.21 ± 2.21	139.05 ± 3.19	106.80 ± 6.27^a^
G5	40.82 ± 0.41	126.89 ± 1.69	109.77 ± 0.69^a^

Values shown are mean ± SD (n = 6). Mean differences are significant (P < 0.05) when compared with: ^a^G1 (control group), ^b^ G2 (cadmium only)

### Effect of *M. myristica* on the levels of liver glutathione and activities of liver catalase and superoxide dismutase

A significant reduction (P < 0.05) was observed in the hepatic enzymatic and non-enzymatic antioxidants (GSH, CAT and SOD) in the cadmium treated group (G2) when compared with the negative control (G1). GSH level as well as SOD and CAT activities were significantly enhanced in the treatment groups (G3 and G4). The result of the extract treated group is comparable with that of the Livolin forte treated group (G5) (Table 4).

**Table 4:** The effect of *M. myristica* on the levels of liver glutathione and activities of liver catalase and superoxide dismutase

Treatment group	GSH (nmole/mg protein)	CAT (unit/min/mg protein)	SOD (unit/min/mg protein)
G1	36.42 ± 0.52^b^	65.83 ± 5.43^b^	27.04 ± 0.05^b^
G2	23.92 ± 0.37^a^	42.73 ± 0.69^a^	15.79 ± 0.06^a^
G3	37.78 ± 0.38	59.66 ± 0.65	24.99 ± 0.13
G4	44.28 ± 0.40^a^	64.31 ± 0.79	35.02 ± 0.08
G5	41.74 ± 0.61^a^	68.02 ± 0.18	31.37 ± 0.79

Values shown are mean ± SD (n = 6). Mean differences are significant (P < 0.05) when compared with: ^a^G1 (control group), ^b^ G2 (cadmium only)

### Effect of *M. myristica* on liver histopathological examination

**Fig. 1:**
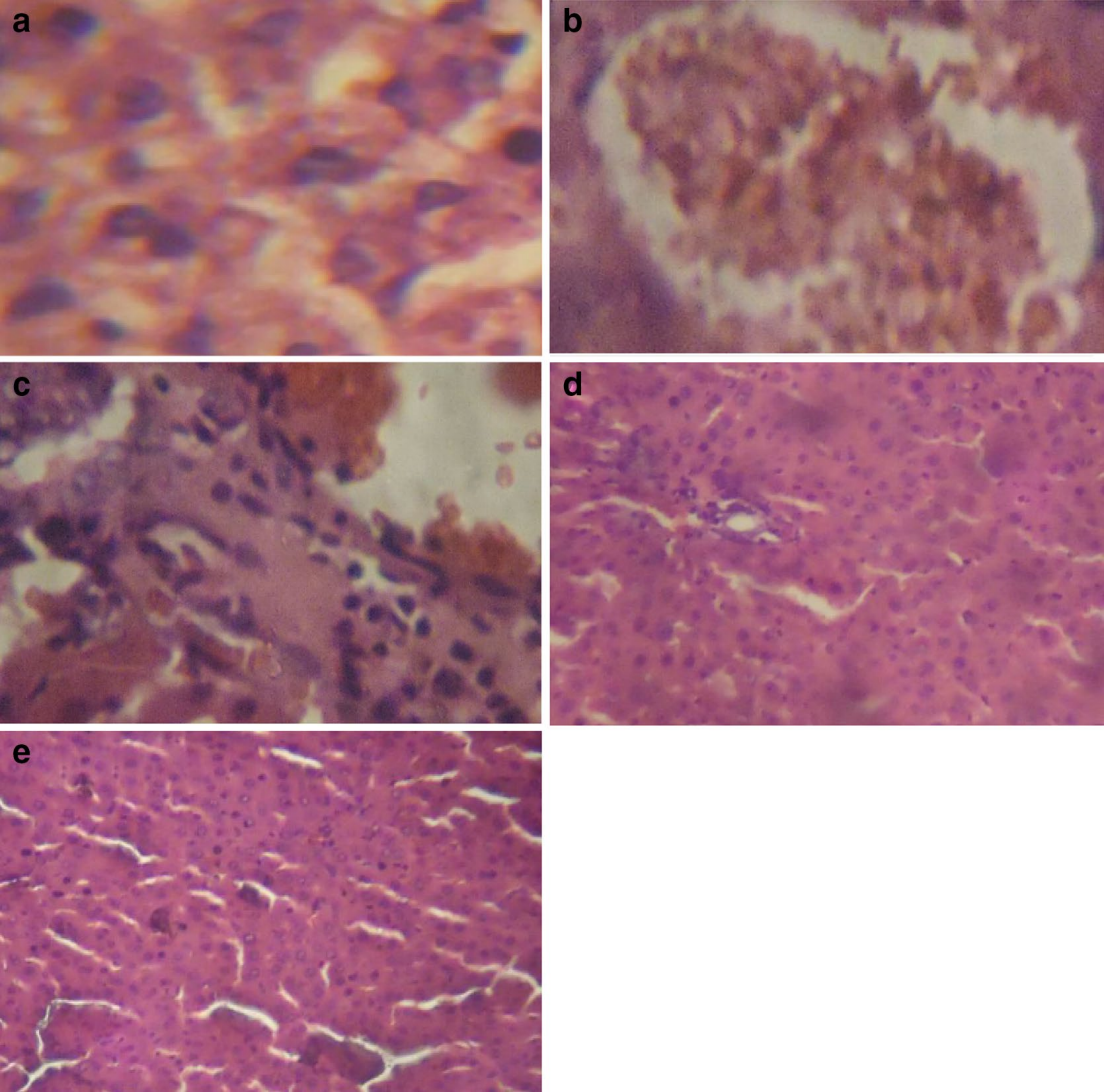
Histological examination of rat livers stained with hematoxylin and eosin (H&E) (×400). **a** G1: showing no abnormal morphological alteration, **b** G2: showing extensive morphological disruption, **c** G3: showing moderate degeneration of hepatocytes and kupper cells, **d** G4: showing mild periportal hepatic necrosis hepatic, **e** G5: showing very mild degeneration of hepatocytes and kupper cells

The histological investigation of the liver tissue (Fig. 1) showed no abnormal morphological alteration in the control group (G1). However, there was extensive morphological disruption in group 2 (G2), characterised by inflammation with focal hepatocytes destruction. In group 3 (G3), moderate degeneration of hepatocytes and kupper cells was observed while group 4 (G4) showed mild periportal hepatic necrosis hepatic. It is interesting to note that group 5 (G5) demonstrated liver tissue with very mild degeneration of hepatocytes and kupper cells.

## Discussion

The present study established that administration of aqueous extract of *M. myristica* (200 or 400 mg/kg bw) significantly protect the liver against toxicity exerted by cadmium. The synergetic effects of the bioactive constituents present in the extract may be responsible for the observed results. This observation complemented previously identified protective roles of antioxidant supplements in amelioration of cadmium-induced hepatocellular damage. Cadmium is one of the most harmful heavy metal that is extensively utilized in diverse industrial processes because of its unique chemical and physical properties, for example it is used in television screens, lasers, batteries, paint pigments, cosmetics, as well as in galvanizing steel and as a barrier in nuclear fission (Bernhoft 2013). Its contamination is known to promote induction of oxidative stress, which leads to the progression of severe pathological conditions after prolonged retention in tissues (Baba et al. 2013; Matović et al. 2011).

Basically, cadmium accumulates mainly in the kidney and liver; these two organs are critical targets for acute cadmium toxicity; about 60 % of the entire cadmium that enters the body is deposited in the liver and in the kidneys (this is distributed equally between these two organs; i.e. 30 % in the liver and 30 % in the kidney) while the remaining 40 % is distributed throughout the body. It has an estimated clearance half-life of 25 years (Bernhoft 2013; Zhai et al. 2013). Cadmium induced toxicity is believed to be multifactorial in nature, even though the exact underlying mechanism of action is not completely understood (Jaishankar et al. 2014). In spite of these, cadmium-induced liver damage has been explained using various mechanisms; notable among these mechanisms is its interaction with essential sub-cellular sites such as mitochondria, peroxisomes as well as microsomes. This interaction enhances the generation of free radicals and lipid peroxidation expressed as malondialdehyde: MDA (Matović et al. 2011).

Besides, cadmium is a non-redox metal, therefore its capable of indirectly eliciting oxidative damage to the liver by depleting cellular antioxidant levels especially glutathione as well as depleting protein-bound sulfhydryl groups; which promotes the generation of reactive oxygen species (ROS) such as superoxide ion, hydroxyl radicals and hydrogen peroxide (Wang et al. 2015; Baba et al. 2013; El-Refaiy and Eissa 2013; Renugadevi and Prabu 2010). Cadmium-induced liver damage is believed to be connected with the interactions of these ROS with cellular biomolecules, which alters numerous cellular functions, such as enzyme activities, gene expression and DNA repair mechanisms as well as signal transduction and causes a shift in overall cell redox state. Added to this, cadmium competes with essential metals such as zinc, selenium, copper and calcium, thereby interfering with various cellular processes such as metal membrane transport and energy metabolism (Arroyo et al. 2012).

Liver injury subsequent to cadmium-toxicity is usually established by high levels of serum hepatic marker enzymes indicating cellular leakage and damage of functional integrity of liver membrane architecture. Elevated levels of alanine transaminase and aspartate transaminase are vital parameters to identify liver damage (Gupta et al. 2004). High levels of serum alkaline phosphatase (ALP) are also linked to the condition and function of liver cells. An elevated serum alkaline phosphatase is associated with liver damage (Renugadevi and Prabu 2010). Administration of aqueous extract of *M. myristica* (200 mg/kg or 400 mg/kg bw) mitigated cadmium-induced hepatotoxicity as revealed by the decreased levels of ALP, AST and ALT compared with the cadmium only group: G2 (Asagba et al. 2007; El-Demerdash et al. 2004). The reversal effect observed in this study suggests that MM can provide protection by stabilizing cell membrane in liver damage associated with cadmium.

Documented scientific evidence shows that cadmium interaction with bio-molecules initiates lipid peroxidation, which leads to oxidative stress associated with various cellular damages (Nazima et al. 2015; Asagba et al. 2007). A direct relationship exists between the level of tissue impairment and the amount of malondialdehyde (MDA) produced (Ayala et al. 2014). Hence, the level of MDA can be utilized as an index of peroxidative damage in vivo and the assessment of the vulnerability of tissues to oxidative stress. Therefore, the elevated level of MDA in the liver of cadmium-treated rats is an evidence of increased membrane lipid peroxidation; this observation is in agreement with earlier findings (Asagba et al. 2007; Ding et al. 2013). The reduction in MDA levels in the liver of groups treated with the extract in comparison with Cd-exposed group suggests the ability of MM extract to mitigate cadmium-induced lipid peroxidation.

In like manner, the elevated levels of triglyceride and total cholesterol could be attributed to the harmful effects of cadmium on cell membrane and disruption in lipid metabolism resulting in elevation of hepatic synthesis of triglyceride and/or a reduced clearance rate of triglyceride-rich lipoproteins (Afolabi et al. 2012). This alteration in levels of triglyceride and total cholesterol was significantly attenuated when the extract was administered. Reduced glutathione (GSH) is an essential constituent of the endogenous antioxidant defence mechanism and it functions as direct free-radical scavenger as well as reduces intracellular reactive oxygen species: GSH thereby protects the cell against toxicity and disease (Romão et al. 2006). Cadmium interacts with this cellular biomolecule and use up endogenous GSH as well as protein bound sulfhydryl groups, causing enhanced production of ROS like hydrogen peroxide, hydroxyl radicals and superoxide (Shukla and Kumar 2009). The diminished level of liver GSH in Cd-treated rats in the present study might be due to its reductive defence role in maintaining an oxidant/antioxidant balance during cadmium-toxicity.

SOD and CAT are crucial component of cellular antioxidant defence system, they are important for evading oxidative stress. The significant reduction in the levels of SOD and CAT in the cadmium group may be accredited to a devastating oxidative alteration of enzymatic proteins and biomembrane lipids by reactive oxygen species. Additionally, cadmium has been shown to directly inhibit SOD and CAT activities through Cd–enzyme interaction resulting in perturbation of enzyme topography important for catalytic activity (Obioha et al. 2009). Treatment with different doses of MM extract reversed these changes. The overall result suggests that the extract acts in a similar mode to that of Livolin forte (Olukiran et al. 2014). Histopathological alterations observed in the liver section also support our biochemical findings.

## Conclusion

Conclusively, our results suggest that aqueous extract of *M. myristica* possess strong antioxidants which can ameliorate hepatocellular damage caused by cadmium intoxication in experimental rats in a dose dependent manner by improving the antioxidant defence systems as well as mitigating lipid peroxidation associated with cadmium toxicity.

## Methods

### Plant material and preparation of extract

Seeds of *M. myristica* were purchased from a local market in Ibadan, Nigeria. Identification and authentication of the seeds were previously carried out by Nwozo et al. 2015. Thereafter, the seeds were de-hulled, air-dried at room temperature and milled into fine powder with a laboratory blender. One kilogram of the powder was defatted in 2 L of n-hexane using a magnetic stirrer at room temperature. The mixture obtained was filtered and 600 g of the residue was air-dried at room temperature. The air-dried residue was macerated in 1.5 L of distilled water for 72 h. Subsequently, the extract was filtered using a clean muslin cloth and Whatman No. 1 filter paper; the filtrate was concentrated by evaporating it to dryness using a rotary evaporator at 60 °C to constant mass, yielding a brown extract. The yield of the preparations was 6.38 % which was kept in a sterilized sample bottle at 4 °C until when needed.

### Reagents and chemicals

All kits used in this study were from Randox Laboratories, Ardmore, Co. Antrim, UK, while the chemicals and reagents were purchased from Sigma Chemical (St Louis, MO, USA) and Merck (Germany).

### Experimental animals

Thirty male Wistar albino rats (weighing 200 ± 20 g) raised in the animal house of Afe Babalola University were used for the experiment. Animals were housed in polypropylene cages and kept at 24 ± 2 °C under 12:12 h of light and dark cycle for acclimatization. The animals had free access to standard pellet diet and water ad libitum. Thereafter, the animals were divided into five groups (G1–G5), G1 was maintained on tap water only and served as the negative control while G2–G5 were maintained on cadmium (200 mg/L Cd as CdCl_2_) in the animals’ main drinking water for 21 days to induce hepatotoxicity. G2 served as the positive control (cadmium only), G3 and G4 were treated with aqueous extracts of *M. myristica* (MM) at doses of 200 and 400 mg/kg bw respectively while G5 was treated with 20 mg/kg bw of Livolin forte (LF). Animal experimental procedures were in conformation with the protocols for the Care and Use of Laboratory Animals as stipulated by the National Institutes of Health (NIH Publication No. 85-23 revised 1985). The Institutional Animal Care and Use Committee of Afe Babalola University also approved this protocol.

**Table:** 

Groups	Treatment
G1	Negative control (tap water only)
G2	Positive control (cadmium water only)
G3	Cadmium water + *M. myristica* (200 mg/kg bw)
G4	Cadmium water + *M. myristica* (400 mg/kg bw)
G5	Cadmium water + Livolin forte (20 mg/kg bw)

### Phytochemical analysis

Phytochemical screening of the seeds of *M. myristica* was done using standard method of Harborne (1973).

## Biochemical analysis and histological examination of tissues

### Preparation of tissues for biochemical analysis and histological examination

Subsequent to the 21 days daily exposure, the animals were sacrificed 24 h after the last dose by cervical dislocation. Thereafter, blood samples were then collected by retro-orbital puncture and allowed to coagulate at room temperature for 30 min. Serum was then obtained by blood centrifugation at 3000 rpm for 10 min and stored at 20 °C until needed for assays. Liver samples were rapidly expunged and washed in ice-cold 1.15 % KCl solution, dried out with filter paper and weighed. Thereafter, portions of the rats’ liver were homogenized in four volumes of 56 mM Tris–HCl buffer (pH 7.4) comprising 1.15 % KCl, and then centrifuged at 10,000*g* for 15 min. The supernatant was collected and kept till required for assays. The other liver portions were preserved in 10 % formalin and used for histological assessment of the liver. Histology was carried out by a qualified pathologist having no prior knowledge of the group the rat livers belonged to. This method allowed for unbiased description of the histological lesions, which were, present or absent in the samples. The liver tissues were stained with haematoxylin and eosin (H & E). The stained tissue sections were thereafter observed under a light microscope (400× objective) for histological assessment.

### Biochemical assay

Serum Alkaline phosphatase (ALP), alanine aminotransferases (ALT) and aspartate aminotransferases (AST) were determined according to the method of Reitman and Frankel (1957), cholesterol and triglyceride (TG) were measured spectrophotometrically using Randox commercial assay kits. Lipid peroxidation measured by malondialdehyde (MDA) content was assayed by the thiobarbituric acid method of Varshney and Kale (1990). The levels of reduced glutathione (GSH) in the supernatant fraction of the liver homogenate were assessed using the method described by Beutler (1963). Catalase (CAT) activity was determined by measuring the rate of decomposition of hydrogen peroxide at 570 nm as described by Sinha (1971). SOD activity was determined using the method of Misra and Fridovich (1972).

### Statistical analysis

Values were expressed as the mean ± SD of six animals. Data were analysed with one-way analysis of variance (ANOVA). Thereafter, the post hoc Duncan multiple tests for analysing biochemical data using SPSS (10.0) statistical software were done. P values <0.05 were considered statistically significant.
